# Optimizing Radiology Workflow: A Multilevel Approach From Department Design to Workstation Ergonomics

**DOI:** 10.7759/cureus.109717

**Published:** 2026-05-27

**Authors:** Andrea Molinari, Florina Feta, Stefania Russo

**Affiliations:** 1 Diagnostic Imaging, Sanremo Hospital (Ospedale di Sanremo), Local Health Authority 1 (Area Sanitaria Locale 1), Sanremo, ITA

**Keywords:** healthcare environment, radiology department design, radiology workflow, reading room design, workstation ergonomics

## Abstract

Radiological practice is characterized by a high cognitive workload and is influenced by environmental and organizational factors. This narrative review, based on a structured PubMed literature search, evaluates the role of radiology department design in shaping clinical workflow and improving service quality. Evidence supports a multilevel approach to department design. Structured layouts separating patient, mixed, and staff-only areas can optimize workflow, while “sterile reading rooms” reduce interruptions and improve diagnostic performance. Patient-centered interventions, including optimized waiting environments and clear informational materials, reduce anxiety and improve care perception. Radiologist-focused strategies, such as ergonomic workstations and optimized lighting, help reduce fatigue and musculoskeletal disorders. In conclusion, integrating architectural, environmental, and ergonomic interventions can improve workflow efficiency, patient experience, and diagnostic safety.

## Introduction and background

Radiological practice is characterized by a high cognitive workload and is not solely dependent on individual factors; rather, it is strongly influenced by the environmental and organizational context in which clinical activity takes place. This includes noisy environments, uncomfortable and non-ergonomic workstations, as well as frequent interruptions that disrupt concentration. These conditions can readily lead to diagnostic errors and radiologist frustration, which may ultimately impact the quality of care and the patient experience [1,2]. A prospective study evaluating daytime weekday reporting shifts identified an interruption approximately every 12 minutes, with a mean interruption duration of about 2.5 minutes, a timeframe considered sufficient to negatively affect concentration during cognitively demanding tasks [1].

These challenges have led to the introduction of mitigation strategies, including supportive initiatives such as mindfulness programs and interventions aimed at improving workplace well-being [3]. In addition, institutions are increasingly focusing on enhancing the patient experience beyond clinical care alone, with a growing trend toward a holistic approach and environments designed according to patient- and family-friendly principles [4].

This work aims to provide a narrative review of the influence of departmental design, analyzing its impact on radiological workflow and offering practical recommendations to improve efficiency and overall service quality.

Within this framework, we propose three main levels of intervention: a macroscopic level, related to architectural planning and radiology department design; a patient-oriented level, aimed at optimizing the patient experience and reducing stressors that may affect interactions with healthcare staff; and a radiologist-oriented level, focused on the working conditions of the radiologist, including ergonomic, environmental, and organizational aspects of the workstation.

Methods

A narrative review of the literature was conducted to evaluate the impact of environmental and organizational design on radiological practice. The review was structured into three primary thematic domains: (1) radiology department architecture and design, (2) patient-oriented interventions, and (3) radiologist-oriented interventions (ergonomics and workstation design).

A structured literature search was performed using the PubMed database. The search was limited to articles published within the last 10 years (2016-2026) and restricted to the English language. The search strategy employed a combination of Medical Subject Headings (MeSH) and free-text terms using Boolean operators (AND, OR). To ensure a comprehensive overview, the search was divided into three specific strings corresponding to the thematic domains (Table 1).

**Table 1:** PubMed search strategy, identified records, and articles included in the qualitative synthesis The table reports the PubMed search strings used for each thematic domain, the number of records identified, the final number of articles included after title/abstract screening and qualitative full-text assessment, and the additional sources identified through targeted manual searching (“snowballing” and direct search engine queries). As this work was designed as a narrative review rather than a systematic review, the search process was not intended to represent a formal PRISMA-based study selection workflow.

Domain	PubMed search string	Filters	Records identified	Articles included in qualitative synthesis
Architecture/department design	(“radiology department” OR “imaging department” OR “medical imaging department”) AND (“hospital design” OR “facility design” OR “department layout” OR “hospital planning” OR architecture)	English; 2016–2026; Abstract	144	8
Patient-oriented interventions	(“waiting room” OR “waiting area” OR “healthcare environment” OR “hospital environment”) AND (“patient anxiety” OR stress OR “patient experience” OR “patient satisfaction” OR “well-being”)	English; 2016–2026; Abstract	546	9
Radiologist-oriented interventions	(“radiology workstation” OR “radiology reading room” OR “reporting room” OR PACS) AND (ergonomics OR “human factors” OR lighting OR interruptions OR hygiene OR “infection control”)	English; 2016–2026; Abstract	86 (1 duplicate)	6

For the architectural domain, search terms included combinations of “radiology department,” “hospital planning,” and “facility design.” Studies were selected when they provided architectural layouts or expert perspectives from architects or designers.

For patient-centered interventions, the search focused on environmental factors influencing patient anxiety and stress in waiting areas, using combinations of terms related to anxiety, waiting environments, and hospital settings. Studies were included if they evaluated environmental or structural interventions with potential applicability to radiology departments.

For radiologist-oriented factors, the search included terms such as “radiology workstation,” “reading room,” “PACS,” and “ergonomics” or “human factors.” Studies addressing workstation design, lighting conditions, hygiene, and infection control in radiological environments were considered.

Titles and abstracts were screened for relevance to the study topics. Additional targeted sources were identified through manual screening of reference lists of key articles and direct search engine queries, regardless of the publication date filter, because of their conceptual relevance. Eligible studies were grouped according to the domains of interest. The inclusion and exclusion criteria applied to each domain are summarized in Table 2.

**Table 2:** Summary of inclusion and exclusion criteria used for article selection in the three investigated domains

Eligibility/Field	Design	Patient	Radiologist
Inclusion criteria	Floor plan or spatial layout; expert opinions from architects or healthcare systems planners	Healthcare waiting rooms; assessing patient outcomes, such as well-being, anxiety levels, or psychological status	Workstation ergonomics; reporting room environment; lighting conditions; workstation hygiene
Exclusion criteria	Organizational or management planning without architectural relevance; not specifically related to radiology departments	Outside healthcare settings; waiting periods related to surgical or invasive procedures	Generic computer workstations not specific to healthcare; interventions not applicable to general radiology practice

Following the screening process, the selected articles were qualitatively analyzed. Data were extracted regarding the intervention type (architectural, patient-oriented​​​​​​, or radiologist-oriented) and the reported impact on clinical practice or well-being. The findings were synthesized narratively to provide a comprehensive overview of the current state of environmental design in radiology and interpreted within a real-world context.

Reporting and methodological transparency were guided by the Scale for the Assessment of Narrative Review Articles (SANRA) [5]. The SANRA items considered most applicable to our predominantly qualitative, rather than statistical, narrative review - including justification of the article’s relevance for the readership, clear definition of the review aims, transparency of the literature search, and appropriate referencing - were independently self-assessed by each author and subsequently discussed jointly to ensure at least an adequate standard across all items.

## Review

Architecture

A well-designed healthcare environment can significantly improve both patient experience and staff performance. Traditionally, architectural design has been considered a secondary and subjective factor; however, the concept of evidence-based design has progressively shifted this perspective by promoting design choices grounded in scientific evidence to optimize clinical outcomes and healthcare workers' well-being [4].

In radiology, these principles have been further developed into architectural models to support cognitive work. Among these, the Eudaimonia Machine, proposed by architect David Dewane and later adapted at The Children’s Hospital of Philadelphia, represents a conceptual framework designed to facilitate “deep work,” defined as sustained states of concentration, productivity, and creativity in environments increasingly affected by interruptions [6].

This model is based on a sequence of spaces with progressively decreasing levels of distraction, ranging from open, interaction-oriented environments to highly protected areas dedicated to cognitively demanding tasks. Such spatial progression enables a gradual transition toward deeper focus while supporting operator well-being and performance [6].

Full implementation of this model may be easier in newly designed departments or during major renovations. Indeed, in the study by Larsen et al., the proposed layout was evaluated using a full-scale mock-up environment housed within the hospital property [6]. Experimental studies aimed at modifying the overall architectural structure of a radiology department to generate higher levels of evidence, therefore, require substantial economic resources, time, and logistical effort, particularly in departments housing large imaging equipment, where temporary structural modifications are not always feasible or easily reversible. Nevertheless, several core principles of the model can still be progressively adapted to existing radiology settings through incremental organizational and environmental interventions.

**Figure 1:**
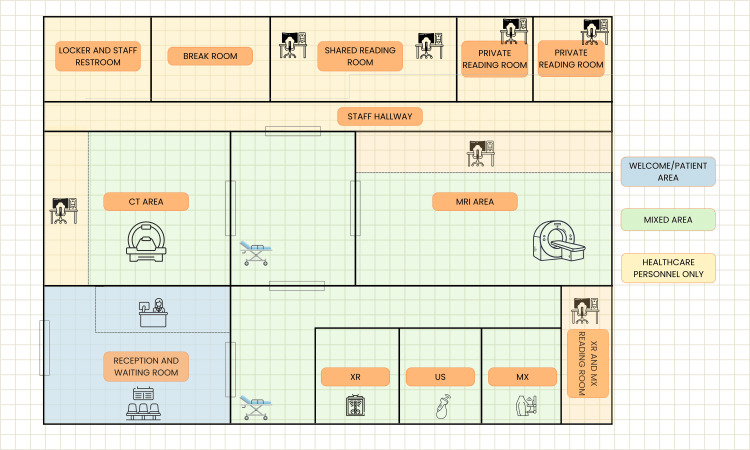
Department layout This image was created using Canva (Canva Pty Ltd., Sydney, Australia). CT: Computed tomography; MRI: Magnetic resonance imaging; XR: X-ray; US: Ultrasound; MX: Mammography.

In the context of a small general radiology department, we propose a simplified three-level functional model reflecting a transition from public to controlled-access spaces: a welcome/patient area, a mixed area, and a staff-only area (Figure 1).

The "welcome* *area" represents the initial point of contact with the patient and is dedicated to reception and waiting, including administrative spaces and waiting rooms designed to reduce anxiety and improve patient experience. Interactions with healthcare staff are limited to structured and essential encounters.

The "mixed area" serves as the operational interface between patients and diagnostic activities. It includes access pathways to "light" imaging modalities (ultrasound, conventional radiography, and mammography), primarily for outpatient services, as well as connections to high-complexity imaging areas such as computed tomography and magnetic resonance imaging. These spaces are designed to accommodate higher patient flow, accessibility for stretcher transport, and increased staff and equipment movement.

The "staff-only area" consists of spaces exclusively reserved for healthcare personnel, organized around a dedicated "staff hallway" that allows internal circulation without overlap with patient pathways. This area includes both shared and individual reporting rooms. Individual rooms enable the application of the “sterile cockpit” principle, providing low-distraction environments that support diagnostic accuracy. Additional support spaces, such as changing rooms, restrooms, and break areas, contribute to staff well-being and long-term sustainability.

A key feature of this model is the presence of dedicated pathways allowing staff direct access to CT and MRI areas from the staff-only zone, as well as efficient internal communication routes via the mixed area, minimizing interference with patient flows.

The organization of shared reading rooms is also critical, as radiological workflow involves frequent interactions with clinicians, technologists, and support staff. Optimal design should ensure a clear separation between consultation and reporting activities, enabling radiologists to select the most appropriate environment and reducing cognitive interruptions.

This model is intended as a conceptual and organizational framework rather than a detailed architectural plan. Technical spaces, ancillary services, and precise spatial dimensions are beyond the scope of this work, which focuses instead on functional layout and workflow optimization.

Sterile reading room

The reporting room can be considered the core architectural unit of radiological practice, where diagnostic interpretation is translated into the final radiological report. Radiological practice involves complex and prolonged cognitive tasks in which sustained attention plays a central role. Frequent interruptions during image interpretation negatively impact performance, reducing efficiency and increasing the risk of diagnostic error. Over time, workflow fragmentation may lead to cumulative inefficiencies, increased cognitive load, and a higher risk of burnout [6].

Observational studies indicate that a substantial portion of a radiologist’s working time is occupied by noninterpretative tasks and interruptions, directly affecting reporting turnaround times. Phone calls, clinical consultations, and administrative duties - often performed concurrently with diagnostic activities - divert cognitive resources away from image interpretation. Moreover, each interruption requires task resumption and refocusing, further increasing mental workload and reducing efficiency [7,8].

Report turnaround time is a performance indicator of radiology department efficiency and is influenced not only by workflow organization but also by environmental design [9]. In this context, workspace configuration plays a crucial role in modulating the frequency and impact of interruptions [10].

A study conducted in a Brazilian university hospital demonstrated that the implementation of subspecialty-based reporting cells in physically isolated areas reduced interruptions by separating reporting activities from high-traffic, noisy environments [10]. At the same time, shared reporting rooms among radiologists with similar expertise facilitated collaboration and consultation. This led to a hybrid model integrating “sterile” reading rooms, designed to maximize concentration, with shared spaces that support controlled interaction [10].

The concept of minimizing nonessential interruptions finds a parallel in the aviation industry, one of the most advanced sectors in terms of operational safety [11]. The “sterile cockpit rule” mandates that, during critical phases of flight (e.g., take-off and landing), all nonessential activities and communications are suspended to reduce the risk of human error, and cabin crew are not allowed to enter the cabin [12].

A similar principle can be applied to radiology, where reporting represents a critical phase of the diagnostic process. The introduction of protected reporting environments (“sterile reading rooms”), characterized by restricted nonurgent interruptions, controlled access, and reduced environmental stimuli, may improve concentration and diagnostic safety.

From an organizational perspective, this requires protocols that clearly distinguish between urgent and deferrable communications, as well as the possibility for radiologists to select work environments based on the level of concentration required. Structurally, these spaces should ensure optimal conditions, including noise reduction and minimal external interference, while maintaining functional proximity to clinical services in emergency settings.

One possible implementation strategy could involve allowing routine communication between technologists and radiologists during scheduled image acquisition activities (“cruising phase”), while protecting the later reporting phase, when radiologists may dedicate focused time to the completion or revision of more complex cases - a phase conceptually comparable to the “take-off and landing” periods in aviation sterile cockpit protocols.

Reducing interruptions during reporting should therefore be considered a key strategy for optimizing radiology workflow and enhancing patient safety, in line with practices adopted in other high-risk systems [12].

Waiting room

The waiting room represents the primary point of contact between the patient and the radiology department and plays an important role in modulating perceived anxiety [13]. Waiting times, although often scheduled, may vary due to daily operational factors, contributing to patient discomfort. Several environmental interventions have been shown to improve patient experience and reduce the subjective perception of waiting.

In general, studies conducted in waiting rooms within general healthcare environments, as well as before ambulatory surgical or dental procedures, suggest that low-impact visual and auditory stimuli - such as television programs, informational content, or nonintrusive background music - may contribute to a more relaxing atmosphere [14,15]. Similarly, the use of decorative elements, including artwork depicting natural landscapes or neutral scenes, contributes to a reassuring visual environment while avoiding emotionally distressing content [16].

Providing educational materials, such as posters or informational leaflets, may further enhance patient engagement. In radiology, information regarding radiation safety and dose awareness can improve patient understanding and perceived quality of care [17].

Comfort-related factors also play an important role. Adequate seating, access to magazines, and internet connectivity contribute to an overall more positive experience, reducing the perception of impersonality often associated with healthcare environments [18].

Additional studies have highlighted the benefits of multisensory interventions, including olfactory stimuli (e.g., aromatherapy), natural elements such as plants, and access to outdoor spaces or well-designed interiors [9,13,19,20].

Evidence also supports the integration of visual art, such as paintings, murals, sculptures, and photography, in healthcare settings, with positive effects on both patients and staff [21]. Notably, such interventions have been associated not only with improved subjective well-being but also with measurable physiological benefits, including reductions in blood pressure and heart rate [13].

These elements are generally low cost. An Italian study demonstrated that even simple interventions, such as the creation of a photographic gallery in a hospital ward, can positively influence patient acceptance of hospitalization [22].

Patient journey

The patient experience in radiology should not be considered in isolation but rather as part of the broader healthcare pathway within the hospital setting. The design and organization of a radiology department are closely interconnected with overall hospital planning and the perceived quality of healthcare services [23].

Various categories of patients may access the waiting and triage areas of the radiology department, including ambulatory outpatients, stretcher-bound patients, hospitalized patients with relative autonomy, and critically ill patients requiring staff assistance, life-support devices, or continuous monitoring systems.

At the time of arrival, outpatients and any accompanying caregivers have often already formed an initial impression based on multiple factors, including ease of access to the facility, availability of parking, clarity of signage, adequacy of information, and overall environmental conditions [24]. Deficiencies in these areas may increase stress levels and negatively influence the perception of care, which can subsequently affect interactions with radiology staff [25].

Patients who arrive in a state of discomfort or frustration may exhibit increased emotional reactivity, potentially impacting communication and the overall quality of the radiological experience. Therefore, in addition to department-specific interventions, it is essential to consider broader structural and organizational factors, such as accessibility, availability of ancillary services (e.g., cafeterias), efficiency of front-desk operations, clarity of wayfinding, and general environmental maintenance.

Overall, the patient experience should be conceptualized as a continuous journey, in which each phase, from hospital access to examination, contributes significantly to perceived quality of care and engagement with the healthcare system.

Ergonomics and lighting

During a typical working day, radiologists may spend more than eight hours at the workstation, continuously interacting with keyboards and mice for image management and interpretation [6]. This prolonged use, combined with repetitive movements, is associated with a high prevalence of musculoskeletal disorders: approximately one-third of radiologists develop overuse syndromes, such as carpal or cubital tunnel syndrome, as well as neck and lower back pain [26]. Furthermore, more than half of radiologists report experiencing repetitive strain injuries, and up to 55% acknowledge maintaining inadequate posture for prolonged periods during daily work activities [26].

Several interventions already adopted in clinical practice, such as ergonomic adjustment of seating, desk height optimization, the ability to modify monitor height and tilt, and the use of more ergonomic input devices (e.g., alternative mice and keyboards, including gaming-derived devices), represent a concrete first step toward improving working conditions. Attention to environmental factors, such as temperature control, further contributes to overall comfort [26,27].

An often overlooked aspect is lighting, which is frequently considered only after the workspace layout has already been finalized, thereby limiting the possibility of subsequent modifications. Notably, a survey conducted by the American College of Radiology (ACR) highlighted that appropriate lighting is considered by radiologists to be a key priority for workplace well-being, comparable to noise reduction [26].

Lighting represents a critical factor not only for diagnostic perception but also as a potential occupational risk. Current workplace health and safety regulations, particularly the Italian Legislative Decree 81/2008, classify lighting among the physical agents requiring risk assessment, similarly to noise and vibrations. These regulations emphasize the need to ensure both adequate general and task-specific illumination, appropriate contrast between the display and the surrounding environment, and the avoidance of glare, excessive reflections, and marked luminance differences [28,29]. Additional guidance is provided by international recommendations, such as those from the American Association of Physicists in Medicine (AAPM) and the European standard EN 12464-1, which define several quantitative lighting parameters, including average illuminance, illuminance uniformity, glare, color rendering index, luminance distribution, and color temperature. Although these parameters allow for an objective assessment of lighting conditions, in clinical practice, attention can be effectively directed toward simple and adaptable solutions (Table 3) [29-31].

**Table 3:** Lighting recommendations for radiologists in reading rooms

Recommendations	Purpose
Avoid reporting in complete darkness.	Reduces excessive contrast between diagnostic monitors and the surrounding environment, potentially limiting visual fatigue.
Prefer soft, indirect background lighting.	Ambient lighting should be indirect and overhead to provide visual comfort while reducing the risk of glare and reflections on diagnostic displays.
Avoid direct light sources facing the monitors.	Helps prevent screen reflections that may interfere with image interpretation.
Consider adjustable backlighting systems.	Backlighting may reduce luminance differences within the radiologist’s visual field and improve perceived visual comfort.
Individualized lighting control.	Enables rapid light adjustment for secondary tasks, such as note-taking or reading printed material.

In this context, the study by Leccese et al. demonstrated that the introduction of an adjustable LED backlighting system can significantly improve visual conditions at radiology workstations [29]. Specifically, the ability to modulate the height, direction, and intensity of light reduces luminance differences between the radiologist’s visual field and the surrounding environment. Luminance, defined as the amount of light perceived from a surface within the visual field, represents a key parameter, as excessive differences between the monitor and the surrounding environment increase visual strain.

The study also reported that most radiologists experienced a subjective improvement in working conditions and a reduction in visual fatigue following the adoption of an adjustable backlighting system [29]. In this context, an optimal reading environment may be characterized by reduced ambient lighting combined with diffuse, indirect, and adjustable light sources, ensuring luminance uniformity and minimizing visual contrast [29]. Overall, lighting optimization represents a high-impact intervention that can improve visual comfort, reduce fatigue, and indirectly enhance diagnostic performance [29].

Hygiene

The hygiene of radiology workstations represents a frequently underestimated aspect of daily clinical practice. Hospital cleaning services, often organized according to general protocols, tend not to systematically address electronic equipment, partly due to concerns about damaging sensitive devices or incurring liability. As a result, the cleaning of keyboards, mice, microphones, and work surfaces is often delegated to healthcare staff or, in some cases, physicians themselves.

Evidence has demonstrated that medical workstations can serve as significant microbiological reservoirs, particularly with regard to high-contact devices such as keyboards and dictation microphones [32].

In particular, a study by Duszak et al. showed that contamination levels of voice recognition microphones were higher than those found on toilet seats and door handles [33]. Mean colony counts (colony forming unit (CFU)) were 69 for dictation microphones, 10.5 for toilet seats, and 14.8 for bathroom door handles [34]. The contamination was primarily due to *Staphylococci* (including *Staphylococcus aureus* and other species) and Gram-negative enteric bacteria [33].

Voice recognition microphones are especially critical, as they are used in close proximity to the airways and often have irregular surfaces that make effective cleaning more difficult. Furthermore, contamination is facilitated by the shared use of workstations among multiple physicians.

The need to preserve the integrity of electronic equipment also limits the use of aggressive disinfectants or large volumes of liquid, contributing to suboptimal sanitation practices [34].

In this regard, the adoption of simple and low-cost measures, such as the use of disposable microphone covers, may represent a practical and effective solution. These devices, like other commonly used consumables in clinical settings (e.g., gloves and masks), can help reduce the risk of cross-contamination without significantly impacting costs or workflow. Alternatively, proper hand hygiene before workstation use, followed by surface disinfection, may be equally effective [33].

Limitations

This work is subject to the inherent limitations and potential biases of a non-systematic narrative review, including possible subjectivity in source selection and incomplete retrieval of the available literature. Moreover, the literature search was restricted to a single database (PubMed), potentially limiting the comprehensiveness of the review. Furthermore, the organizational and architectural proposals discussed in this study are presented from a conceptual perspective and have not always undergone prospective or experimental validation within a specific radiology setting.

## Conclusions

Inadequate planning of the radiology department at both macro- and micro-levels may contribute to diagnostic errors, compromise patient safety, and negatively affect workflow efficiency. A multilevel approach enables effective design strategies, with architectural and organizational interventions requiring institutional involvement, while radiologist- and patient-centered solutions can be implemented locally at relatively low cost and with significant impact on quality and staff well-being. Although radiologists may lack formal training in architectural design, their understanding of workflow and clinical needs supports their active involvement in planning processes. Expanding the literature on radiology workplace design beyond traditional engineering and architectural perspectives may better reflect clinical practice and promote institutional awareness and engagement. Looking ahead, prospective studies assessing environmental and workflow-related interventions in relation to objectively measurable outcomes, such as interruption times, may help shape future evidence-based radiology workplace design.
